# Specific IgA Enhances the Transcytosis and Excretion of Hepatitis A Virus

**DOI:** 10.1038/srep21855

**Published:** 2016-02-25

**Authors:** Natalie A. Counihan, David A. Anderson

**Affiliations:** 1 Centre for Biomedical Research, Burnet Institute, Melbourne, Victoria, Australia

## Abstract

Hepatitis A virus (HAV) replicates in the liver, and is excreted from the body in feces. However, the mechanisms of HAV transport from hepatocytes to the gastrointestinal tract are poorly understood, mainly due to lack of suitable *in vitro* models. Here, we use a polarized hepatic cell line and *in vivo* models to demonstrate vectorial transport of HAV from hepatocytes into bile via the apical cell membrane. Although this transport is specific for HAV, the rate of fecal excretion in inefficient, accounting for less than 1% of input virus from the bloodstream per hour. However, we also found that the rate of HAV excretion was enhanced in the presence of HAV-specific IgA. Using mice lacking the polymeric IgA receptor (pIgR^−/−^), we show that a proportion of HAV:IgA complexes are transported via the pIgR demonstrating a role for specific antibody in pathogen excretion.

Hepatitis A virus (HAV) is an orally transmitted hepatotropic picornavirus that first infects the mucosa of the gastrointestinal tract. Once this epithelium is penetrated, blood-derived HAV presumably reaches the liver via portal circulation. Antigen is first detected in liver-residing Kupffer cells 1–3 weeks post experimental inoculation, followed shortly by infection of hepatocytes^1^,^2^,^3^. Viremia is present from 2 weeks post-infection, and can persist for 4 weeks to over a year^1^,^4^. Following extensive replication in hepatocytes, HAV is excreted from the body in feces, but the mechanism of transport remains ambiguous. Interestingly, HAV circulating in blood is closely associated with a lipid membrane, perhaps as an antibody-evasion mechanism, but virus shed in feces lacks this membrane for reasons unknown^5^. In order to better understand HAV trafficking pathogenesis, suitable *in vitro* models are required to reflect the highly polarized nature of intestinal and hepatic tissue.

Cell polarity is a property of epithelial tissue, and is achieved by the asymmetrical expression of proteins on apical (luminal) and basolateral (vascular) surfaces. This cellular orientation and organization is fundamental for the physiological functions of mucosal and hepatic tissues, including the absorption and secretion of hormones, lipids and proteins. In hepatocytes, the canalicular membrane serves as the apical pole and leads directly into the biliary canaliculus. Thus, any substrate exported from this membrane may traverse the gastrointestinal tract in bile. Basolateral cargo are exported into the space of Disse and the hepatic sinusoids which have extensive connectivity to blood vessels.

There are several *in vitro* models of polarized tissue including MDCK and Caco-2 cells, both of which display simple, columnar orientation of polarity amenable to *in vitro* manipulations. However, these non-hepatic models are not appropriate for studies with hepatotropic viruses, as hepatocytes have unique and complex mechanisms for polarized transport. In addition, hepatocytes do not display columnar orientation of polarity that is typical of mucosal tissue. Rather, they display a complex three-dimensional structure that is not amenable to *in vitro* culture using normal methods. Further, primary hepatocytes are technically difficult to grow, and rapidly lose their polarization upon culture^6^. A clone of HepG2 cells have been identified that maintains the functional characteristics of hepatocytes, yet displays the morphology typical of columnar epithelia, representing the first *in vitro* model of polarized hepatocytes^7^. This HepG2-derived N6 clone of cells was used to investigate trafficking of HAV from polarized hepatocytes. It was shown that progeny HAV was almost exclusively exported via the basolateral membrane, suggesting that infected hepatocytes excrete virus into the blood rather than the bile as expected. Whilst these finding readily explain the viremia observed in the actuate phase of the disease, they do not account for high titer virus shed in feces. Thus a fundamental question about HAV pathogenesis still remains: how does the virus reach the gastrointestinal tract for enteric excretion? In this paper we investigate a mechanism for HAV export using N6 cells as a model for polarized hepatocytes.

Infection with HAV induces both IgG and IgM antibodies, but also a prolonged IgA response^2^,^8^. IgA is an essential component of mucosal immunity, and is transported through cells via a well-characterized secretory pathway via a specific receptor, the polymeric immunoglobulin receptor (pIgR). This type I transmembrane protein has an affinity for IgM or polymeric IgA (pIgA). IgA can function to prevent attachment of pathogens, participate in intracellular neutralization of viruses, and assist in immune elimination by clearing antigen complexes from systemic circulation (reviewed in^9^). Blood to bile transport of IgA through hepatocytes has been described for rats and mice^10^,^11^,^12^, but studies in humans using a suitable *in vitro* model have been inconclusive^13^,^14^,^15^,^16^,^17^.

IgA has long been implicated in HAV infection. HAV-specific IgA has been detected in the feces and serum of infected hosts as early as 4 days after the onset of symptoms, and can persist for three years or longer^18^,^19^,^20^,^21^. Additionally, HAV-specific IgA has been partially associated with virus purified from feces^2^,^8^, thus may be implicated in virus excretion. Considering the close association of IgA with HAV infection, we sought to investigate the contribution of IgA to HAV transport and export. Taking into account the normal secretory pathway of IgA is in the basolateral to apical direction, we hypothesized that blood to bile transport of HAV may be enhanced in the presence of specific IgA. The aim of this study was to explore the mechanism for HAV export from hepatocytes, and to determine the role of specific IgA in this process using both *in vitro* and *in vivo* models.

## Results

### HAV is trafficked from basolateral to apical membranes *in vitro*

In this study, we used a previously characterised model of columnar polarized hepatocytes as *an in vitro* model to investigate transcytosis of HAV.

**Figure 1:**
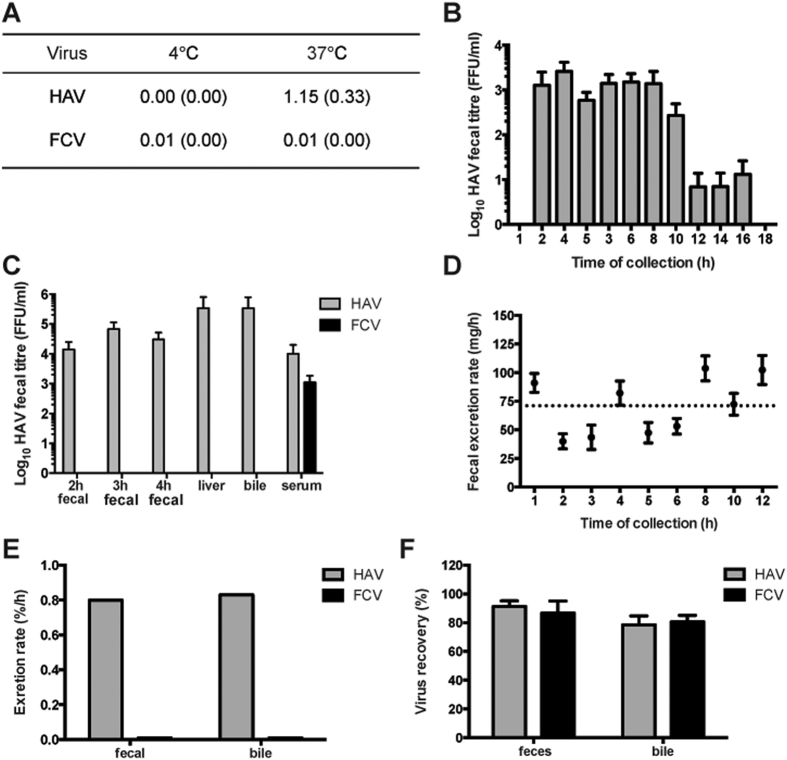
HAV is specifically trafficked from basolateral to apical membranes. (**A**) *In vitro* trafficking of HAV in polarized N6 cells. HAV and FCV were added to the basolateral compartment of polarized cultures, incubated at the indicated temperature and apical supernatants titrated for infectivity after 2 h. Data show mean rate of transport as a percentage of input virus+/− SEM (n = 3 (FCV) or 12 (HAV). (**B**) *In vivo* transcytosis of HAV. To demonstrate blood to bile transport, feces were collected from HAV-injected mice and the indicated time point post-injection and the HAV titer determined. Data show mean HAV titer+/− SEM (n = 4). (**C**) Transcytosis is specific for HAV. To compare transport of HAV and FCV, groups of mice were injected with either virus and samples collected at the indicated time points. Data show average viral titers of HAV (grey) and FCV (black)+/− SEM (n = 6). (**D**) Average excretion rates of C57/Black6 mice. Feces were collected from age- and species-matched mice at the indicated times to determine fecal excretion rates. Data show average weight at each time+/− SEM (n = 6). The dotted line shows the mean weight across all timepoints. (**E**) Excretion rates of HAV and FCV. The rate of HAV (grey) and FCV (black) excreted in bile and feces was determined from 4 h samples. Data show mean rate of transcytosis as a percentage of input virus (n = 6). (**F**) Recovery of HAV and FCV in feces and bile. Samples were obtained from non-injected control mice and spiked with equal amounts (10^6^ FFU or PFU) of HAV (grey) and FCV (black). Samples were incubated at 4 °C and titrated to determine percentage recovery+/− SEM (n = 4).

To measure active transport of virus in the basolateral to apical direction, 10^6^ focus-forming units (FFU) of HAV was added to the basolateral side of cultures and incubated at 37 °C or 4 °C for 2 h. In addition to HAV, feline calicivirus (FCV) was included in the same inocula as a control for non-specific viral transport. The most basolateral to apical transport was observed in HAV cultures that were warmed to 37 °C, suggesting active transport of HAV (Fig. 1A).

### HAV is trafficked from basolateral to apical membranes *in vivo*

*In vitro* results indicate that HAV shows polarized movement towards the apical pole by a temperature-sensitive mechanism. Although these findings were obtained using a polarized cell line, they cannot be directly translated into *in vivo* situations, as cell polarity within the liver is invariably more complex than our cell culture model. Additionally, it is difficult to quantify the proportion of completely polarized cells, thus results vary considerably between experiments.

To overcome these limitations, these experiments were extended using an *in vivo* system. A mouse model was selected as they are not the natural hosts for HAV, thus can be used to study virus transport independent of infection^22^ and have previously been used to study basolateral to apical transport of hepatic IgA^10^,^23^. Virus can be delivered directly to basolateral membranes via intravenous (iv) injection of the tail vein. It was hypothesized that HAV would be trafficked to the apical pole of hepatocytes, secreted with bile into the gastrointestinal tract, and then excreted in feces. To investigate, mice were injected with HAV and at regular intervals fecal samples were collected and titrated for HAV infectivity. Significant quantities of HAV were recovered from all samples collected between 2 and 16 hours post-injection (Fig. 1B). As peak virus titers were observed after 4 h, subsequent experiments were restricted to this time frame. To investigate specificity of basolateral to apical virus transport, feces were collected from mice injected with either HAV or FCV and virus titers determined (Fig. 1C). Bile and blood samples were also collected for HAV titration and processed as described in Materials and Methods. Basolateral to apical trafficking was specific to HAV, as FCV was not detected in bile or fecal samples (Fig. 1C). Importantly, the serum concentrations of FCV and HAV were not significantly different (p = 0.5281), confirming that each group received comparable inocula.

### Fecal and biliary excretion rates of HAV and FCV

In Fig. 1C, fecal virus titers show mean tires per ml of virus, which equates to 1g of feces. To determine the rate at which virus was excreted from mice, the average mouse defecation rate per hour was determined over a 12h period (71 mg feces/h; Fig. 1D) and virus titers were multiplied by this figure to determine the amount of virus excreted per hour. This was then expressed as a percentage of input virus (Fig. 1E). The amount of bile excreted per mouse was calculated using bile excretion rates previously published (305 μl/100 g of body weight per hour^24^) Individual mice were weighed and their weight-adjusted bile excretion rate determined. This rate was then used to calculate biliary excretion rates per hour, which was then expressed as a percentage of input virus (Fig. 1E). Excretion of HAV from feces and bile were strikingly similar (0.80–0.83% of input virus per hour), with limited FCV detected. To ensure the lack of FCV in feces and bile was not due to inactivation, samples were spiked with known quantities of HAV and FCV and close to all the input HAV and FCV was recovered (Fig. 1F). Together, the data from Fig. 1 supports the hypothesis that HAV is transported from the basolateral to apical membranes *in vitro* and *in vivo* (although somewhat inefficiently), via a specific, temperature-dependent mechanism of transcytosis. These data represent evidence of HAV being excreted from hepatocytes via an indirect mechanism.

### Characterization of HAV-specific IgA antibodies

**Figure 2:**
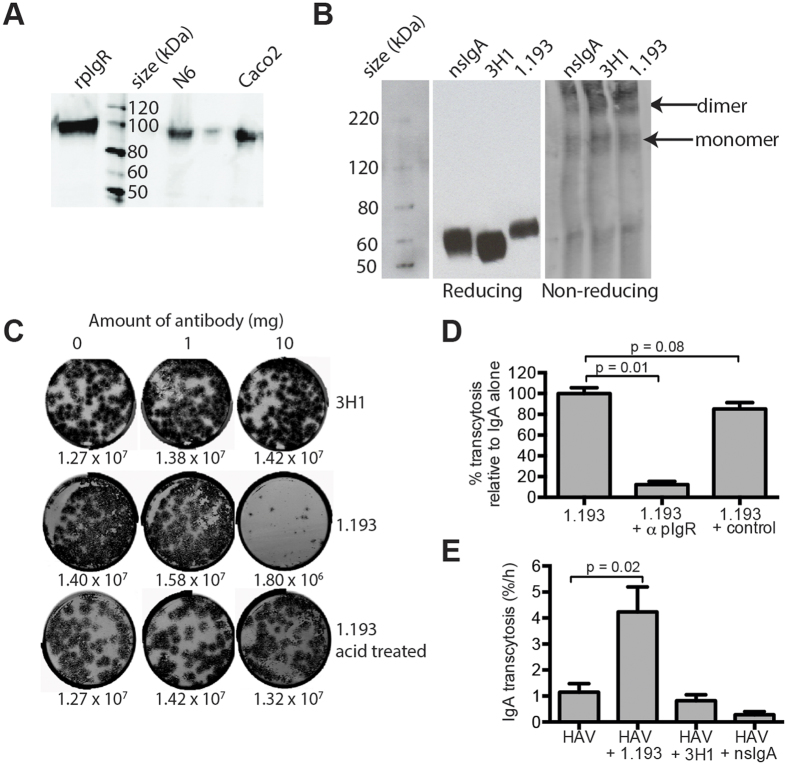
Transcytosis of IgA. (**A**) Western blot of pIgR from N6 and Caco2 cells. To confirm protein expression of pIgR, lysates of N6 and Caco-2 cells were probed with pIgR-specific antibody; recombinant pIgR (rpIgR) served as a positive control. A single specific band of similar size to rpIgR was observed for both cell types. (**B**) Separation of IgA MAbs. HAV antibodies and nsIgA were subjected to electrophoresis under reducing and non-reducing conditions. Monomeric and dimeric IgA were observed for all 3 antibodies. (**C**) Neutralization of IgA antibodies. Equal amounts of HAV were incubated with indicated amounts of antibody and titrated for infectivity by FIFA. Foci of infection were counted to determine HAV infectivity, and are shown for each sample in FFU/ml. 1.193 samples were acid treated to prior to titration recover infectious virus. (**D**) 1.193 transcytosis is inhibited with anti-pIgR antibody. Basolateral membranes were pre-incubated with 2.5 μg of anti-pIgR antibody or isotype control for 1 h. 1.193 was then added and the rate of transcytosis determined after 1 h. The reduction in IgA transcytosis with antibody was determined relative to no treatment (n = 3). (**E**) *In vitro* HAV transcytosis is enhanced with 1.193 antibody. Virus-antibody complexes were incubated, and then added to the basolateral membrane of polarized cultures. Supernatants were collected after 2 h and titrated for HAV infectivity. 1.193-containing supernatants were acid-treated prior to titration (n = 5–10). For both graphs, error bars show SEM and statistical significance was determined using an unpaired t-test.

IgA has a strong association with HAV, and can enhance infection of enterocytes^8^,^25^. Additionally, IgA is transported through epithelia into secretions in the same direction as HAV (basolateral to apical) to transport IgA from the vascular system into bile. Due to the close association of IgA with HAV and their common intracellular trafficking pathways, we investigated the effect of specific IgA on HAV transcytosis. pIgR-mediated transcytosis of IgA has been extensively studied as a model for basolateral to apical transcytosis, however, the majority of data was obtained using MDCK cells transfected with rabbit pIgR^26^,^27^. IgA transport mechanisms have been described for rodent hepatocytes but parallel studies with humans are less well described and localization of human pIgR was difficult to demonstrate in non-polarized cells^13^,^15^,^16^,^17^. Investigations of hepatic IgA transport were performed using our polarized N6 model of hepatocytes. Before investigating transcytosis, we first confirmed the presence of pIgR in polarized N6 cells by immunoblotting (Fig. 2A) using Caco2 cells and recombinant pIgR (rpIgR) as positive controls. A species similar in size to rpIgR representing full length pIgR was observed in N6 and Caco2 cells, but not irrelevant BSC-1 cells (data not shown). RT PCR was used to confirm mRNA expression, and validated by DNA sequencing (data not shown). Once pIgR expression was confirmed, HAV-specific IgA antibodies were sourced to investigate IgA-mediated trafficking of HAV including a previously characterised anti-HAV monoclonal antibody 1.193^28^,^29^. An additional IgA antibody was induced by immunizing mice with inactivated HAV (see Materials and Methods). Both antibodies were reactive to HAV antigen by ELISA (data not shown) and contained significant proportions of dimeric IgA and higher order structures (Fig. 2B, about 50% dimeric IgA). Under reducing conditions, a single band representing IgA heavy chains was observed for each antibody. To determine the neutralizing capacity of each antibody, equal amounts were mixed with virus for 1 h, and then titrated for infectivity (Fig. 2C). 1.193 antibody was found to be a neutralizing antibody, and infectious virus could only be recovered after acid treatment (see Materials and Methods).

### *In vitro* transcytosis of IgA

To demonstrate transcytosis of IgA, HAV antibody 1.193 was added basolaterally to polarized cultures and titrated for IgA by ELISA. In 3 experiments, an average of 1.53% (+/− SEM of 0.27) of input antibody was detected in the apical compartment after 1 h compared with 0.27% (+/− SEM of 0.03) of dextran. These results indicate that 1.193 is transported preferentially over dextran, but somewhat inefficiently, perhaps due to 1.193 containing a significant amount of monomeric IgA. To determine if pIgR was responsible for this transport, pIgR was blocked by pre-incubation with anti-pIgR antibody and the rate of 1.193 transcytosis determined (Fig. 2D). Addition of anti-pIgR antibody reduced IgA transcytosis to <15% of non-treated samples, suggesting that a significant amount of transcytosed IgA is mediated by pIgR. 3H1 and 1.193 antibodies were next complexed with HAV and added to basolateral compartments and the rate of basolateral to apical transport of HAV determined (Fig. 2E). For these experiments, non-specific IgA (nsIgA) was used as a control. Results indicate that the most efficient trafficking of HAV was observed when virus was complexed with 1.193 antibody (p = 0.02). There was no significant difference in the amount of HAV present when complexed with either 3H1 or nsIgA.

### HAV fecal excretion is enhanced with IgA

**Figure 3:**
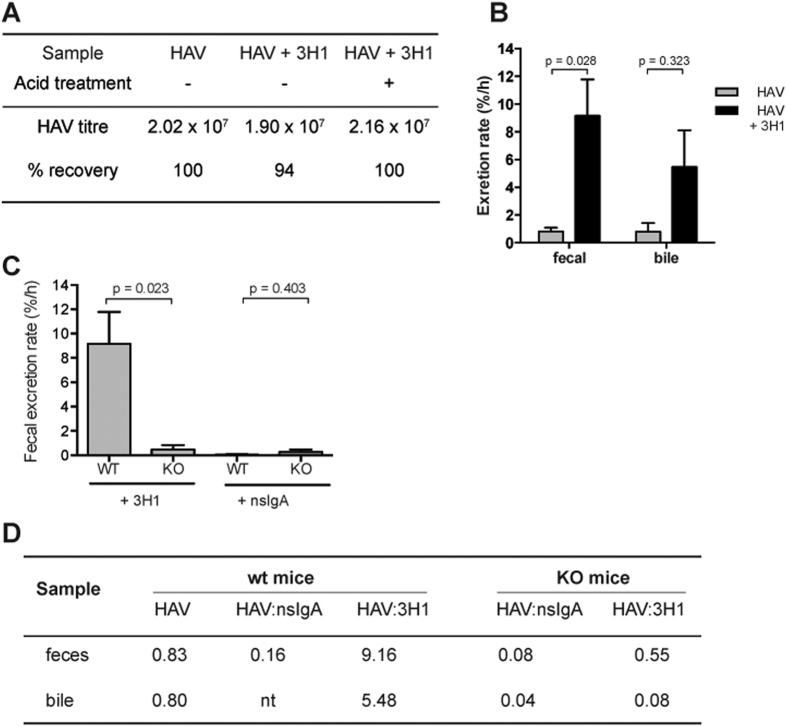
*In vivo* transcytosis of HAV is enhanced in the presence of specific IgA and is mediated by pIgR. (**A**) HAV can be recovered from fecal samples spiked with HAV:3H1 complexes. Fecal samples were spiked with HAV:3H1 complexes and HAV recovered with and without acid treatment. Data show average HAV titers after recovery and recovery rate when compared with pre-treatment samples. (**B**) *In vivo* transcytosis of HAV:3H1. The mean rate of HAV excretion in mice injected with HAV (grey) or HAV:3H1 complexes (black)was determined after 4 h from feces and bile. (**C**) HAV:IgA transcytosis is mediated by pIgR. The rate of HAV fecal excretion was determined in WT and KO mice injected with either HAV:3H1 or HAV:nsIgA complexes. For both graphs, n = 6, error bars show SEM and an unpaired t-test was used to compare rates of excretion. (**D**) Rates of HAV transcytosis. Excretion rates for all groups of mice at 4 h are compared (nt = not tested).

As results from *in vitro* experiments demonstrated enhanced transcytosis with specific antibody (Fig. 2E), trafficking of HAV:IgA complexes was next investigated *in vivo* to see if transcytosis efficiency was altered in the presence of specific IgA. Pre-incubated HAV:IgA complexes were delivered to the liver of C57/Black6 mice via iv injection. In pilot experiments, recovery of virus from HAV: 1.193 complexes was poor due to the neutralizing nature of this antibody despite treatment with acid to disrupt the complex (<5% of input virus was recovered, data not shown). However, HAV was efficiently recovered from fecal samples spiked with 3H1 both with and without acid treatment (Fig. 3A), so this antibody was used for subsequent experiments. Feces and bile were collected 4 h post-injection and excretion rates calculated using previously described methods. Interestingly, the amount of HAV recovered from feces of mice receiving complexed virus was significantly higher than those receiving virus alone, but these effects were not significant in bile (Fig. 3B).

### HAV:IgA export is mediated by IgA secretory pathway

To further investigate HAV transcytosis with IgA, knockout mice (KO) lacking the IgA receptor (pIgR^−/−^) were used^30^. As these mice do not express a functional receptor for IgA in intestinal or hepatic tissues, IgA transcytosis is reduced^30^,^31^. Despite lacking a receptor, minimal amounts of IgA were detected in feces and bile (Supp Fig. 1). To investigate HAV transcytosis, groups of WT and KO mice were injected with HAV complexed with either nsIgA or 3H1 and feces collected after 4 h (Fig. 3C). KO mice receiving HAV:3H1 complexes had a significantly lower rate of HAV transcytosis compared with WT mice. Figure 3D shows bile excretion rates for KO mice, and summarises all the transcytosis experiments presented in Figs 1, 2, 3. HAV alone is transcytosed in feces at a peak rate of 0.83% of virus per hour, but this is increased about 11-fold with the addition of 3H1, and similar trends are observed for bile. When compared with WT mice, KO mice show a significant reduction in biliary 3H1 transcytosis (0.08% per hour, compared with 5.48% per hour), but smaller differences are observed in feces. Similarly, in HAV: 3H1-injected WT mice, comparable amounts of HAV were excreted in bile and feces, but KO mice show a marked reduction of HAV secretion in bile compared with feces. It must be noted that the excretion rate of virus complexed to nsIgA injected into WT mice (0.16) and KO mice (0.08) was unexpectedly lower than that of virus alone (0.83%). As it is not likely that HAV is forming complexes with nsIgA, the reduced excretion rate may be due to steric hindrance from the IgA, or due to the sample sizes used in the experiments (n = 6).

## Discussion

The work described here provides experimental evidence of HAV secretion into feces. Interestingly, HAV is first sorted to the basolateral membrane prior to export at the apical pole^7^, a seemingly complicated route for secretion, but consistent with default sorting pathway of hepatocytes^32^,^33^,^34^,^35^,^36^. The presence of virus at the basolateral membrane may readily explain the viremia observed during acute illness^1^,^37^. A recent study has described two distinct populations of HAV that exist during infection; exosome-like enveloped HAV circulating in blood, and non-enveloped virus that is shed in feces^5^. The observation of two distinct viral species is interesting, and has implications for the current study. We hypothesize that virus secreted basolaterally from hepatocytes^7^ is a mixture of enveloped and non-enveloped virus and that the pool of naked virus is transcytosed for secretion into bile and excretion in feces. The remaining enveloped virus pool is then able to circulate in blood to facilitate spread protected from neutralising antibody. One limitation of our study is that the viral inoculum that contains a mixture of enveloped and non-enveloped virus^5^ was delivered to hepatocytes via the blood, and not as basolaterally secreted virus, but this was unavoidable.

In this study, we observed blood to bile transport of HAV but fecal excretion rates were low, thus may not account for the large amount of virus shed in feces. The apparent inefficiency of HAV export observed in our system prompted us to further investigate virus transcytosis. We hypothesized that virus may be transported more efficiently via a carrier-mediated mechanism, as has been suggested for HAV export from enteric epithelia^29^. We first investigated IgA as a potential candidate for enhancing HAV export, as this ligand is transported from basolateral to apical membranes in epithelial cells, including hepatocytes. Additionally, increased accumulation of HAV RNA is observed in the liver when the virus is complexed with IgA compared to uncomplexed virus^22^. Finally, HAV-specific IgA is associated with HAV in virus in feces, suggesting a role for IgA in HAV infection^2^,^8^,^38^.

Having demonstrated hepatic transcytosis of IgA, we next investigated HAV:IgA transcytosis. Using *in vitro* and *in vivo* models, we show that fecal virus excretion is enhanced from 0.83% to 9.16% in the presence of specific antibody, but no enhancement was observed with control antibody (Fig. 3D). Studies using pIgR^−/−^ mice showed limited transcytosis of HAV:IgA (Fig. 3C,D), suggesting that HAV transport in pIgR-dependent. Results suggest that HAV may be transcytosed through hepatocytes to the apical pole via 2 mechanisms, via the default pIgR-mediated pathway, and by a pIgR-independent mechanism. Further work needs to be completed to investigate these mechanisms further.

Export of HAV from hepatocytes was inefficient in our hands, although a significant increase in fecal virus was observed in the presence of IgA. The normal function of IgA is to limit pathogen invasion and spread, commonly by preventing attachment to epithelia, or by neutralizing intracellular pathogens^39^,^40^,^41^. IgA has previously been shown to aid in the excretion of antigens^42^,^43^,^44^, but this has not been demonstrated for whole viruses. Thus, the findings in this study are interesting, as IgA is implicated in the export of infectious virus bound to virus-specific antibody, thereby facilitating further transmission of the virus. Despite our findings, the contribution of IgA to HAV secretion is still unclear. Although induction of IgA is rapid compared with the IgG response^21^, a significant proportion of HAV is excreted into feces prior to antibody production^45^, thus a considerable amount of virus shedding occurs in the absence of IgA. In one study, a bi-phasic pattern of fecal excretion was observed in 2 out of 3 animal subjects^45^, suggesting that excretion may be enhanced once a robust IgA response is established, however this hypothesis requires further examination. Despite the ambiguity over the contribution of IgA to virus excretion, it is clearly important as fecal HAV has been observed coated with sIgA^8^. Thus our results shed some light on the complex transmission of HAV and present an interesting example of a virus exploiting a cellular mechanism for enhanced transmission.

## Materials and Methods

### Ethics statement

All animal experiments were conducted in accordance with the NHMRC Australian code of practice for the care and use of animals for scientific purposes (7^th^ edition, 2004) and were approved by the AMREP animal ethics committee (project number E/0515/2006/F).

### Culture of polarized cells

Polarized N6 cells were cloned from HepG2-derived C3A cells (American Type Culture Collection) and maintained as previously described^7^. For *in vitro* transcytosis experiments, a single cell suspension containing 5 × 10^4^ N6 cells were seeded onto Transwell-COL porous tissue culture inserts (Costar) with 3.0 μm pore size and incubated at 37 °C for up to 24 days, using Williams medium E and supplemented with 10% FCS, 100 μg/ml streptomycin, 100 U penicillin, 2 mM glutamine, 10 mM tris and 1% dimethyl sulfoxide.

### Assays for cell polarity

After 14 days in culture, cells were tested often to assess polarity using a variety of methods. Human hepatocytes produce albumin that is secreted basolaterally into blood, thus the proportion of albumin present in basolateral supernatants of each culture was used as an indicator of polarity, as has been described previously^7^. Briefly, an ELISA was used to detect human albumin in apical and basolateral chambers. Cultures were deemed polarized if >85% of albumin was basolaterally secreted. Additionally, diffusion of a small molecule (10kDa dextran) was used as a marker of polarity, as tight junction formation in polarized cells should restrict movement of such molecules. The rate of Alexa 680-labelled dextran transport was determined by adding 10 μl to basolateral compartments and regularly sampling apical supernatants. The intensity of infrared fluorescence of triplicate samples was then measured using the Odyssey infrared imager and used to determine the percentage diffusion per hour. Cells were deemed sufficiently polarized if dextran trafficking was <0.30%/hr. Monolayer integrity was also assessed by immunofluorescent staining for tight junction protein Zo-1, as previously described^7^.

### Viruses and infectivity assays

HAV strain HM175A.2 was propagated in BSC-1 cells and purified and quantitated as previously described^7^,^46^. FCV was propagated in CRFK cells and purified and titrated as previously described^7^.

### IgA antibodies

Antibodies utilized included previously characterized anti-HAV IgA 1.193 antibody (kindly provided by Prof. S. Lemon)^47^ and purified non-immune mouse IgA (Sigma). Anti-HAV IgA 3H1 was obtained by immunizing Balb/C mice with two doses of 10^7^ FFU equivalents of formaldehyde-inactivated HAV at four-week intervals via the intraperitoneal route. Mice were bled four weeks post-injection, and sera tested for reactivity by ELISA to detect total antibody. Splenic fusion and hybridoma cloning was performed at The Walter and Eliza Hall Institute (Melbourne, Australia). Supernatants were screened for reactivity by ELISA. Antibody isotype was determined using IsoStrip (Roche).

### Characterization of pIgR and IgA antibodies

To investigate expression of pIgR in N6 and Caco2 cells, clarified lysates were separated by SDS-PAGE and probed with anti-human pIgR (R&D Systems) by western blot. Recombinant pIgR (R&D Systems) was used as a positive control. IgA antibodies were characterized by Western Blot using a 4–15% precast gradient gel under reducing and non-reducing conditions. To determine the neutralizing capacity of IgA antibodies, 1–10 μg of antibody were mixed with HAV for 1 h at 37 °C and titrated for infectivity by FIFA. To recover infectious virus from HAV neutralized with 1.193, complexes were treated at pH 1.5 (using 0.1 M glycine pH 1.0) for 1 h. Post -incubation, complexes were returned to pH 6.0 with 1 M NaOH, and titrated for infectivity. To block pIgR, 2.5 μg of anti-human pIgR or isotype control were incubated in the basolateral compartment for 1 h, then IgA added for transcytosis experiments.

### ELISA for HAV IgA antibody

Maxisorb microtiter plates were coated overnight at 4 °C with 0.1% purified, inactivated HAV antigen (BioDesign) in 0.1 M NaHCO3 (pH 9.6) and blocked with 0.5% casein in PBS. HAV-specific total antibody was detected with anti-mouse HRP. Total IgA was detected with goat anti-mouse IgA HRP. Color development was observed with the addition of tetramethylbenzidine, and stopped with 0.5 M H_2_SO_4_. Optical density was measured at 450 nm and 620 nm.

### *In vitro* transcytosis experiments

For HAV transcytosis experiments, virus was diluted 10-fold in serum-free (SF) media to a final titer of 10^6^ FFU or PFU per well, chloroform extracted and further diluted to 2.6ml total volume with media containing 1% serum. The inoculum was added to the basolateral domain of polarized cultures and incubated at 37 °C or 4 °C for 2 h. Following incubation supernatants were removed and culture replaced with fresh media containing 1% serum. Supernatants were titrated for infectivity by FIFA and plaque assay to determine titers of HAV and FCV, respectively. The rate of HAV or FCV transcytosis was determined as a percentage of input virus per hour. For IgA transcytosis experiments, 10–25 μg of antibody was diluted in 2.6 ml of serum-free media and added to the basolateral compartment of polarized cultures. Supernatants were collected after 2 h to determine the rate of transcytosis. For HAV-IgA transcytosis experiments, 10^6^ FFU of HAV was incubated with 10–25 μg of 1.193, 3H1 or nsIgA for 1 h at 37 °C and then applied to the basolateral membrane of polarized N6 cultures as described previously. Supernatants were titrated for HAV infectivity via FIFA. Supernatants containing 1.193 were acid-treated prior to titration, as described previously.

### *In vivo* transcytosis experiments

To demonstrate transcytosis *in vivo*, 8–10 week old C57/Black 6 (WT) mice were injected iv with 100 μl of virus (10^6^ FFU/PFU of HAV or FCV diluted 10-fold in PBS) via the tail vein^48^,^49^. For experiments with IgA, HAV-antibody mixtures were incubated for 1 h at 37 °C prior to each assay. Infectious HAV was recovered from 1.193-containing samples by acid treatment, as described earlier. In addition, pIgR deficient mice were used in parallel to determine the contribution of IgA to HAV transcytosis. These aged-matched KO mice (pIgR^−/−^) were generated on a C57/Black6 background and were kindly provided by Dr O. Wijburg (University of Melbourne, Melbourne Australia)^30^.

At regular intervals post-injection, fecal samples were collected in PBS +0.1 mg/ml soybean trypsin inhibitor to make a 10% (w/v) suspension, vortexed and spun at 14000 rpm/4 °C/10 min. Supernatants were then frozen until titration. Post-mortem, bile was collected using a 1 ml insulin syringe directly from the gall bladder and diluted 10-fold in SF media. Blood was collected via cardiac bleed, left to clot, spun at 8000 rpm/4 °C/10 min and serum collected. For titration, all samples were diluted ten-fold in media with 1% serum, and titrated for infectivity by FIFA (for HAV) and plaque assay (for FCV). Virus titers for each sample were determined, and expressed as FFU or PFU per ml of sample after correcting for dilutions performed during sample preparation. Using rates of excretion for feces and bile, the amount of HAV and FCV excreted per ml or per gram per hour was determined^24^. It should be noted that these calculations have limitations, as calculations were based on the assumption that each mouse received 100% of the inoculum. However, considering the small inocula size (100 μl), there was most likely variation in the amount of virus delivered to each animal leading to larger variability in experiments. Also, as it was impractical to collect all the feces from each mouse for each timepoints, it was also assumed that the fecal excretion rate estimated in the pilot experiment was applicable to all further experiments.

### Statistical analysis

Statistical analysis (unpaired two-tailed t-test) of data were performed using GraphPad Prism Software. For all *in vitro* data, n refers to the number of times the experiment was independently performed using different cultures.

## Additional Information

**How to cite this article**: Counihan, N. A. and Anderson, D. A. Specific IgA Enhances the Transcytosis and Excretion of Hepatitis A Virus.. *Sci. Rep*. **6**, 21855; doi: 10.1038/srep21855 (2016).

## Supplementary Material

Supplementary Information
