# Supplementary material for: Specific IgA Enhances the Transcytosis and Excretion of Hepatitis A Virus
Source: Sci Rep. 2016 Feb 25;6:21855. doi: 10.1038/srep21855 (PMC4766440; doi:10.1038/srep21855)

Specific IgA Enhances the Transcytosis and Excretion of Hepatitis A Virus

Natalie A Counihan and David A Anderson

Supplementary Figure 1

(A) IgA content of fecal samples in WT and KO mice. Total IgA concentration was determined from pre- and post-injection samples by ELISA for each group of mice. Data show mean IgA concentration (n = 6). (B) IgA content of bile in WT and KO mice. Total IgA was determined in bile samples 4 h after injection. Data show mean IgA concentration (n = 6). For both graphs, previously published normal ranges for both mouse types are shown in parentheses (μg/g) [32]. Unpaired t-tests were used to compare IgA content.

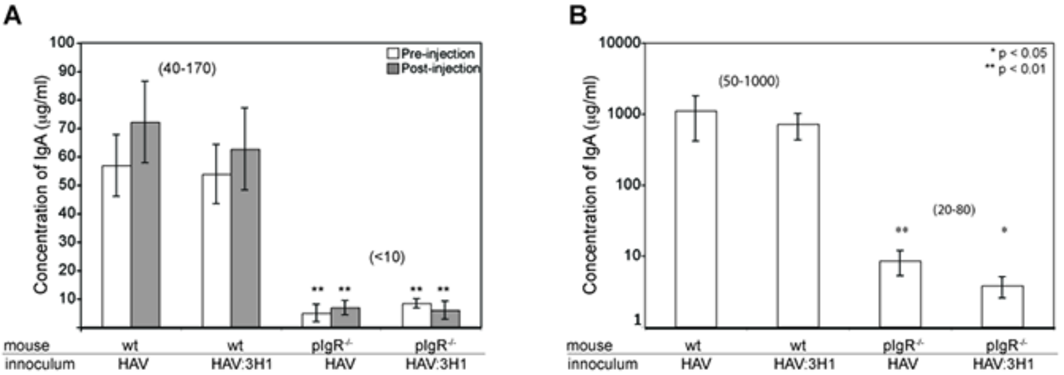

Supplement: Supplementary Information [file srep21855-s1.pdf]
